# Thrombotic Microangiopathy, an Unusual Form of Monoclonal Gammopathy of Renal Significance: Report of 3 Cases and Literature Review

**DOI:** 10.3389/fimmu.2021.780107

**Published:** 2021-11-10

**Authors:** Edward J. Filippone, Eric D. Newman, Li Li, Rakesh Gulati, John L. Farber

**Affiliations:** ^1^ Divsion of Nephrology, Department of Medicine, Sidney Kimmel Medical College at Thomas Jefferson University, Philadelphia, PA, United States; ^2^ Department of Pathology, Sidney Kimmel Medical College at Thomas Jefferson University, Philadelphia, PA, United States

**Keywords:** thrombotic microangiopathy, monoclonal gammopathy of renal significance, eculizumab, plasma cell dyscrasia, C3 glomerulopathies, alternate pathway of complement, atypical hemolytic and uremic syndrome

## Abstract

Monoclonal gammopathies result from neoplastic clones of the B-cell lineage and may cause kidney disease by various mechanisms. When the underlying clone does not meet criteria for a malignancy requiring treatment, the paraprotein is called a monoclonal gammopathy of renal significance (MGRS). One rarely reported kidney lesion associated with benign paraproteins is thrombotic microangiopathy (TMA), provisionally considered as a combination signifying MGRS. Such cases may lack systemic features of TMA, such as a microangiopathic hemolytic anemia, and the disease may be kidney limited. There is no direct deposition of the paraprotein in the kidney, and the presumed mechanism is disordered complement regulation. We report three cases of kidney limited TMA associated with benign paraproteins that had no other detectable cause for the TMA, representing cases of MGRS. Two of the cases are receiving clone directed therapy, and none are receiving eculizumab. We discuss in detail the pathophysiological basis for this possible association. Our approach to therapy involves first ruling out other causes of TMA as well as an underlying B-cell malignancy that would necessitate direct treatment. Otherwise, clone directed therapy should be considered. If refractory to such therapy or the disease is severe and multisystemic, C5 inhibition (eculizumab or ravulizumab) may be indicated as well.

## Introduction

Monoclonal gammopathies result from neoplastic clones of the B-cell lineage (B-lymphocytes, lymphoplasmacytic lymphocytes, or plasma cells) and may cause kidney disease either by direct deposition (1) or indirectly by other mechanisms (2). Direct deposition of immunoglobulin fragments (usually light chains) or whole immunoglobulins may produce well-characterized clinicopathologic entities, such as AL amyloidosis (3), monoclonal immunoglobulin deposition disease (4), and proliferative glomerulonephritis with monoclonal immunoglobulin deposits (5) (see **Table 1**). Indirect mechanisms include disordering alternate pathway of complement (CAP) regulation resulting in C3 glomerulopathy (C3G) (2).

**Table 1:** Monoclonal Gammopathies of Renal Significance (MGRS)*.

**Lesions associated with deposition of the paraprotein (fragment or whole immunoglobulin)**
Light chain cast nephropathy** Light chain proximal tubulopathy, crystalline or non-crystalline subtypes (Cryo)crystaglobulin associated nephropathy Crystal-storing histiocytosis Immunoglobulin-related amyloidosis Monoclonal immunoglobulin deposition disease
Light chain deposition disease Light and heavy chain deposition disease Heavy chain deposition disease
7. Proliferative glomerulonephritis with monoclonal immunoglobulin deposits
8. Cryoglobulinemic glomerulonephritis (Type 1 cryoglobulins)
9. Monoclonal immunotactoid glomerulopathy/fibrillary glomerulonephritis***
**Lesions not associated with paraprotein deposition**
C3 glomerulopathy
C3 glomerulonephritis
Dense deposit disease
2. Thrombotic microangiopathy***

*Refers to specific kidney lesions in patients with paraproteins from B-lineage clones (B-cells, plasma cells, or lymphoplasmocytes) not satisfying criteria for frank malignancy (multiple myeloma, lymphoplasmocytic lymphoma, chronic lymphocytic leukemia, marginal zone lymphoma). The same lesions could be found in patients having such malignancies but are not referred to as MGRS in that situation.

**Rare in the absence of criteria for frank multiple myeloma (where it would be a treatment defining lesion), but possible in which case it would qualify as MGRS.

***Provisional association with paraprotein.

Whereas the tumor burden of neoplastic clones may reach a threshold diagnostic of malignancy, in many circumstances the burden is not large enough to warrant chemotherapy, whether considering myeloma or less aggressive B-lineage malignancies such as chronic lymphatic leukemia or marginal zone lymphoma. In fact, the underlying clone may even be undetectable, and the associated paraprotein is therefore considered a monoclonal gammopathy of undetermined significance (MGUS). When an MGUS is causing kidney disease in the absence of a malignancy requiring treatment, it is now referred to as monoclonal gammopathy of renal significance (MGRS) (6, 7), a diagnosis that warrants clone-directed therapy to prevent progression to end-stage kidney disease (ESKD) and/or recurrence after kidney transplantation if the patient is a transplant candidate.

The thrombotic microangiopathies (TMAs) represent a heterogeneous group of diseases characterized by primary microvascular endothelial cell injury resulting in platelet rich and/or fibrin thrombi occluding small vessels of various organs, mainly kidney and brain (8, 9). Often there is an associated microangiopathic hemolytic anemia (MAHA) and thrombocytopenia indicating a more systemic process. In the absence of MAHA, the diagnosis rests on biopsy, typically of the kidney. The TMAs may be primary (congenital or acquired) or secondary to a variety of drugs or diseases, including various infections (most notably Shiga-toxin producing E coli, STEC), autoimmune diseases, metastatic cancer, malignant hypertension, pregnancy, transplantation (stem cell or solid organ), and other primary glomerulopathies (see **Table 2**). Multiple secondary causes can coexist (10), and secondary causes may also serve as triggers for those with underlying genetic susceptibility to a primary TMA (11). Well characterized primary TMAs include thrombotic thrombocytopenic purpura (TTP), STEC associated hemolytic uremic syndrome (STEC-HUS), and atypical HUS (aHUS). The latter may or may not have a detectable underlying genetic defect in complement regulation, and the C5 inhibitor eculizumab is now approved for aHUS with or without a defined genetic mutation, and it is considered first line therapy (12–14). Additionally, ravulizumab is a humanized monoclonal antibody derived from eculizumab but with a 3X greater half-life requiring dosing every 4 – 8 weeks that is now approved for aHUS (15). There are no trials comparing these 2 C5 inhibitors. Herein, we refer to eculizumab as the prototypical C5 inhibitor with the realization that ravulizumab may be a viable alternative.

**Table 2:** Syndromes of Thrombotic Microangiopathy (TMA)*.

**Primary TMA**
Hereditary
Thrombotic thrombocytopenic purpura (TTP) – biallelic mutations in ADAMTS13
Complement-mediated TMA, also called atypical hemolytic uremic syndrome (aHUS)
Mutations in proteins in the alternate pathway of complement (CAP)**
Metabolism-mediated TMA (homozygous *MMACHC* mutations) – rare
Coagulation-mediated TMA (homozygous *DGKE* mutations) – rare Acquired
Autoantibodies against ADAMTS13
Shiga-toxin-mediated hemolytic uremic syndrome (STEC-HUS)
Autoantibodies against CAP proteins, typically Factor H
Secondary TMA***
Drug-induced TMA
Immune-mediated (e.g., quinine)
Toxic and dose related (e.g., gemcitabine)
Infection-mediated TMA (other than Shiga-toxin producing organisms)
Metastatic cancer
Pregnancy associated TMA, including preeclampsia/HELLP syndrome
Autoimmune associated TMA
Systemic lupus
Antiphospholipid syndrome
Scleroderma renal crisis
Hematopoietic stem cell transplantation associated TMA
Solid organ transplant associated TMA
Paraprotein associated TMA****

*Adapted from George and Nester (8).

**Approximately 50% of patients with a clinical picture compatible with complement-mediated TMA will not have detectable pathogenic mutations in CAP proteins. Some refer to these as idiopathic aHUS, although response to complement inhibition with eculizumab is equally efficacious.

***Many patients with secondary causes have underlying genetic mutations similar to those causing complement-mediated HUS. Such secondary causes may serve in those instances as triggers in predisposed individuals. Treatment should involve removal/treatment of the secondary cause if possible, with consideration of eculizumab on a case by case basis..

ADAMTS13, A disintegrin-like and metalloprotease with thrombospondin type 1 motif, 13; MMACHC, methylmalonic aciduria and homocystinuria type C gene; DGKE, diacylglycerol kinase epsilon gene.

****Provisional.

Current reviews of MGRS list kidney TMA as a relatively rare association (7), and TMA is considered an MGRS lesion having only provisional status (6). The presumption is disordered complement regulation predominantly on the cell surface, caused indirectly by the paraprotein. Direct antibody deposition does not occur and hence is not detectable by immunofluorescence. Since MGUS is relatively common in the elderly, the question arises as to whether this combination is merely the chance association of an MGUS with idiopathic aHUS, as opposed to an acquired cause of a secondary TMA that warrants clone-specific treatment (MGRS). Only a handful of such cases have been reported. We report three well characterized and fully evaluated cases of TMA associated with paraproteins, strengthening the position that this combination has not occurred by chance but represents MGRS and warrants consideration of both clone-specific treatment and possibly eculizumab as well.

## Case Reports

Patient 1, BP, is an 84-year-old man who presented for outpatient evaluation on 6/2/2020 for proteinuria (albumin/creatinine ratio of 1,501 mg/g) and reduced eGFR (serum creatinine 1.68 mg/dl) (see **Table 3**). There was a history of type 2 diabetes, hypertension, hyperlipidemia, and heart failure with preserved ejection fraction. He was receiving apixaban following thromboembolectomy of a femoral artery clot thought to be secondary to atrial fibrillation in a patient not on anticoagulation. Review of symptoms revealed lower extremity swelling and was otherwise unremarkable. There was no constitutional symptoms, purpura, or diarrhea. Physical exam was remarkable for blood pressure 150/50 mm/Hg and lower extremity edema. Serologic evaluation revealed an IgGκ paraprotein detectable in serum, but not in urine.

**Table 3:** Patient laboratory evaluation.

		Patient BP	Patient YM	Patient LB
Initial Laboratory Evaluation	Hemoglobin (g/dL)	12.7 (5/20/2020)	13.5 (3/1/2021)	**5.9** (6/3/2021)
	Platelet count (X10^3^)	201 (5/20/2020)	248 (3/1/2021)	178
	Serum creatinine (mg/dL)	**1.68** (5/20/2020)	0.9 (3/1/2021)	**5.93** (6/3/2021)
	Urine protein/creatinine ratio (mg/g)	**786** (6/2/2020)		**1,900** (6/3/2021)
	Urine albumin/creatinine ratio (mg/g)	**1,501** (2/20/2020)	**1,138** (3/1/2021)	83% of urine protein
	Serum albumin (g/dL)	4.0 (2/20/2020)	4.2 (3/1/2021)	**3.4** (6/3/2021)
	LDH (IU/L)	160	106	142
	Haptoglobin (md/dL)	130	118	236
	Peripheral smear	No schistocytes	No schistocytes	No schistocytes
	ADAMTS13 activity (% normal)		>100%	75%
	C3/C4 (mg/dL)	134/24	168/26	**42**/14
	Factor H antibody (titer)	NA	NA	negative
	Soluble C5b-9 (ng/mL)	191.7	NA	NA
	ANA (titer)	negative	negative	negative
	DRVVT normalized ratio	normal	normal	normal
	Anticardiolipin Antibody (U/mL)	negative	negative	**IgM 21.3** (non-significant)
	Anti-β2-glycoprotein I (U/mL)	negative	negative	NA
	Anti-Scl70 (U/mL)	negative	negative	negative
	SIEP	**IgGκ**	**IgGκ**	**IgMκ**
	UIEP	negative	**IgGκ** (faint)	**Free κ**
	Serum FLC ratio (mg/mg)	1.93	1.27	**8.5**
	Viral PCR (HIV, Hep B/C, CMV, EBV)	EBV 263 copies/ml (normal <200)	Negative or NA	Negative or NA
Most recent Laboratory evaluation	Serum creatinine (mg/dL)	**1.9** (7/1/2021)	**1.37** (8/04/2021)	**3.21** (7/29/2021)
	Urine protein/creatinine ratio (mg/g)	59 (7/1/2021)	111 (8/17/2021)	**2,800** (7/22/2021)
	Hemoglobin (g/dL)	12.6 (7/1/2021)	12.3 (8/04/2021)	**8.0** (7/29/2021)
	Platelet count (X10^3^)	158 (7/1/2021)	229 (8/04/2021)	153 (7/29/2021)
Genetic analysis	C3, Factor H, Factor I, THB	negative	negative	negative

ANA, antinuclear antibody; CMV, cytomegalovirus; DRVVT, dilute Russel viper venom time; EBV, Epstein-Barr virus; FLC, free light chain; LDH, lactate dehydrogenase; NA, not available; PCR, polymerase chain reaction; SIEP, serum immunofixation electrophoresis; THB, thrombomodulin; U, units; UIEP, urine immunofixation electrophoresis.

Bolded values are abnormal, all others are within normal limits.

**Figure 1:**
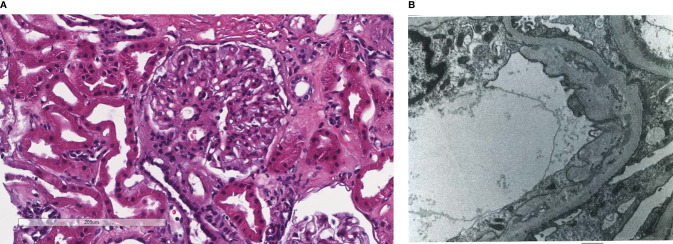
Patient 1. Light microscopy shows an intact glomerulus without hypercellularity or thickened glomerular capillary walls **(A)**. By electron microscopy, there is prominent widening of the subendothelial space by edema, cell debris, and amorphous proteinaceous deposits **(B)**. X10,000.

A kidney biopsy was obtained on 9/30/2020 to differentiate possible diabetic kidney disease (DKD) from MGRS. TMA was diagnosed (see **Table 4** and **Figure 1**) and subsequent assessment for MAHA and secondary causes of TMA was unremarkable. Analysis of the genes for complement Factor H (CFH), complement Factor I (CFI), C3, and thrombomodulin was negative for pathogenic mutations. However, due to financial constraints, we could not assess the genes for membrane cofactor protein (MCP, CD46) or complement Factor B (CFB) for genetic mutations, or the genes for CFH and MCP for at risk haplotypes (vide infra). This same limitation for genetic testing applies to Patients 2 and 3 below. Bone marrow biopsy revealed a 5% plasma cell clone. C5 inhibition (eculizumab or ravulizumab) was not immediately offered since there was no evidence of MAHA and renal function was not acutely deteriorating. Also, the urine protein/creatinine ratio spontaneously improved prior to any treatment (see **Table 3**) and serum creatinine remained stable through 6/2021, precluding a potential C5 inhibitor indication for chronic smoldering disease. Clone directed therapy was initiated with dexamethasone and bortezomib. Despite the apparent clinical stabilization, clone directed therapy was still pursued due to the obvious detectable plasma cell clone and the fear that any relapse could lead to end stage kidney disease. We suspect that the paraprotein in this case was more of a susceptibility factor requiring a “second hit”, perhaps an infection, to manifest disease.

**Table 4:** Pathology of kidney biopsy.

Patient	1	2	3
Light microscopy	thickening of glomerular capillary walls with mesangial expansion and segmental mesangial hypercellularity	Mild thickening of the capillary walls and mild mesangial expansion with segmental mesangial hypercellularity	prominent diffuse diabetic glomerulosclerosis with basement membrane double contours evident in one glomerulus
Immunofluorescence microscopy	negative	negative	negative
Electron microscopy	widening of the subendothelial space by edema, amorphous proteinaceous deposits. Cellular debris, accumulations of basement membrane-like material, and frank reduplications of the basement membraae	prominent and diffuse widening of the subendothelial space by edema, cell debris amorphous proteinaceous deposits, and accumulations of basement membrane-like material; frank reduplications of the basement membrane are present in many capillary loops	diabetic glomerulosclerosis with widening of the subendothelial space by edema, amorphous proteinaceous deposits, cell debris and accumulations of basement membrane-like material

**Figure 2:**
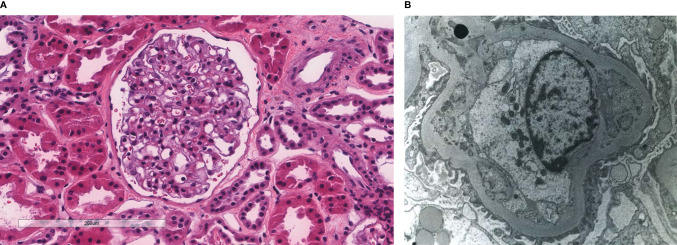
Patient 2. Light microscopy shows an intact glomerulus without hypercellularity or thickened glomerular capillary walls **(A)**. By electron microscopy, there is prominent widening of the subendothelial space by edema, cell debris, and amorphous proteinaceous deposits. Partial basement membrane reduplication is present **(B)**. X8,000.

Patient 2, YM, is a 54-year-old man who presented for outpatient evaluation on 4/1/2021 for albuminuria (albumin/creatinine ratio of 1,138 mg/mg) and normal eGFR (see **Table 3**). There was a 16-year history of type 2 diabetes, hypertension, hyperlipidemia, obstructive sleep apnea, and peripheral arterial disease. There was a history of transient kidney disease as a child, although no biopsy was obtained. Review of systems was unremarkable, including no constitutional symptoms, diarrhea, or cardiovascular symptoms. Initial physical exam revealed a blood pressure of 155/90 mm/Hg, a systolic ejection murmer, and trace edema. Serologic evaluation revealed a low level IgGκ monoclonal protein. A kidney biopsy was obtained on 5/24/2021 to differentiate possible DKD from an MGRS. TMA was diagnosed (see **Table 4** and **Figure 2**) and subsequent assessment for MAHA, underlying genetic mutations, and secondary causes of TMA was unremarkable. Bone marrow biopsy did not reveal a clonal population of cells, and flow cytometry was thought to be unnecessary with the normal marrow and resolving clinical course. Clone-directed therapy was not offered for these reasons. C5 inhibition was not indicated in the absence of MAHA or acute/subacute deterioration of organ function.

**Figure 3:**
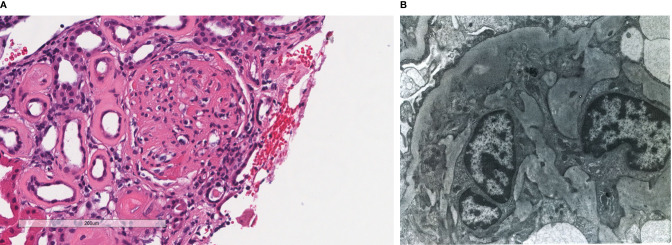
Patient 3. Light microscopy shows an intact glomerulus with prominently thickened capillary walls and segmental mesangial hypercellularity **(A)**. By electron microscopy, there is prominent widening of the subendothelial space by edema, cell debris, and amorphous proteinaceous deposits. Basement membrane reduplication is complete **(B)**. X8,000.

Patient 3, LB, is a 69-year-old man with a 15-year history of type 2 diabetes, hypertension, hyperlipidemia, chronic kidney disease (creatinine 1.9 mg/dl in 3/2020 with 2+ proteinuria on urinalysis) who developed a right groin abscess and was treated with trimethoprim-sulfamethoxazole. After 2 days he presented to the hospital on 6/3/2021 with severe hyperkalemia and acute kidney injury (see **Table 3**). History was remarkable for several months of anorexia and weight loss, daily ibuprofen, and several weeks of loose stools (2 x/day). He was afebrile with BP 140/60, basilar crackles, and trace edema. Following 1 emergency hemodialysis, potassium remained in the normal range and creatinine stabilized at approximately 4 mg/dl. A low level of IgMκ paraprotein was found. Bone marrow biopsy revealed 5% clonal (κ-chain restricted) plasma cells with negative fluorescence in-situ hybridization. A PET/CT scan was negative for any signs of lymphoplasmacytic malignancy. A kidney biopsy was performed on 6/8/2021 (see **Table 4** and **Figure 3**). TMA was diagnosed and subsequent assessment for MAHA, underlying genetic mutations, and secondary causes of TMA was unremarkable. The patient is being treated with clone directed therapy due to the detectable clone and advanced stage of the kidney disease. C5 inhibition was not offered due to the absence of MAHA, the advanced stage of the kidney disease, and the absence of other organ involvement.

## Discussion

We describe 3 cases of biopsy-proven, kidney-limited TMA that had coexisting paraproteins. No secondary cause of TMA was evident upon extensive investigation, and genetic testing revealed no underlying predisposing mutations in the genes that were tested (see Patient 1 above and **Table 3**). In our opinion, this is more than chance association, and the paraproteins are most likely causative. Since none of the 3 patients had a clone of B-lineage cells sufficient to diagnose a malignancy, these cases were classified as MGRS, and 2 of the 3 are being treated with clone-directed therapy (6).

TMA is a pathologic lesion found in a variety of inherited and acquired diseases. The pathology of acute TMA is characterized by non-inflammatory fibrin and/or platelet thrombi occluding capillaries, arterioles, and small arteries with or without fibrinoid necrosis of arteriolar and small artery walls (16, 17). Additionally, endothelial cell swelling and mesangiolysis may be found on kidney biopsy. By electron microscopy (EM), subendothelial widening with electron lucent material is characteristic and is sufficient to make a diagnosis in the absence of characteristic light microscopic findings. All 3 of our patients had characteristic EM findings, confirming the diagnosis of TMA. Chronic pathologic changes by light microscopy include arterial intimal proliferation, often with mucoid changes (“onion-skinning”) and/or hyalinosis, and a double-contoured membranoproliferative pattern on kidney biopsy (17).

The clinical manifestations of these acute and chronic pathologic changes result from ischemic injury of the involved organs, typically kidney and nervous system, but potentially also the gastrointestinal and the cardiovascular systems (18). Frequently, but not always, there is an accompanying MAHA characterized by thrombocytopenia, a Coomb’s negative hemolytic anemia with ≥ 0.5% schistocytes on peripheral blood smear, and other laboratory evidence of intravascular hemolysis, such as elevated lactate dehydrogenase (LDH), absent haptoglobin, and increased indirect bilirubin. Such a MAHA is often the clue to the underlying TMA, and in the appropriate clinical circumstances, it may be sufficient to make a diagnosis in the absence of a biopsy. Patients manifesting a MAHA most likely will be detected when there are acute pathologic changes, and such patients are the ones most in need of immediate and often life-saving therapy. Our patients did not manifest these systemic features of MAHA and had chronic, kidney-limited disease proven by biopsy. Hence, immediate anti-complement therapy was not mandatory.

The underlying pathophysiology of TMA, whether acute or chronic, generally involves endothelial cell injury, typically with activation of platelets, the coagulation cascade, and/or complement, especially *via* the CAP. Evidence for activation of complement by the classic and/or lectin pathways also exists given the frequent positive staining for C4d (19). The causes of TMA are divided into primary and secondary TMA (8). The primary causes may be hereditary or acquired. Hereditary primary causes include TTP, caused by mutations in ADAMTS13, and complement-mediated HUS caused by pathogenic mutations in regulatory/effector proteins of the CAP, such as CFH, CFI, MCP, C3, CFB, and thrombomodulin. At risk haplotypes that enhance TMA susceptibility include the CFH tgtgt (20) and MCP ggaac haplotypes (21), each found in about 3% of the population, and the CFH-related proteins (CFHR) 3 – 1 deletion that results in CFH autoantibodies found in about 8% of the population (22). Genetic abnormalities resulting in disordered metabolism (homozygous mutations in *MMACHC*) or coagulation (mutations in *DKGE*) are additional rare causes of primary genetic TMA. Acquired primary TMA results from antibodies to ADAMTS13 (acquired TTP) or to complement regulatory proteins, especially CFH (23, 24) and possibly CFI (25) (acquired primary complement-mediated aHUS). Additionally, STEC-HUS is considered an acquired primary TMA (8), although it is caused by an infection.

There is no diagnostic test to confirm primary aHUS, and it remains a diagnosis of exclusion after ruling out TTP, STEC-HUS, and secondary causes of TMA. Overall, about 50% of patients considered to have primary aHUS may have detectable mutations and warrant the designation primary complement-mediated aHUS. Hence, about 50% of patients with primary aHUS will not have detectable genetic mutations, and relatives displaying the same mutations as affected patients frequently do not manifest disease. These mutations generally display a dominant inheritance pattern and are usually present in the heterozygous state (26). Penetrance is said to be about 50% (26), although recent data indicate it may be as low as 20% (27). Therefore, pathogenic mutations causing complement-mediated aHUS are in essence susceptibility factors, usually requiring a secondary trigger to produce disease, typically an infection, but also possibly pregnancy, transplantation, or malignant hypertension, and such triggers may be found in 70% of patients (26). Multiple mutations may coexist in the same patient [perhaps 5% - 10% of cases with defined mutations (28)], as may one or more at-risk haplotypes.

Secondary TMA is much more common than the primary TMA (10), and causes/triggers include pregnancy, infections, malignancy, transplantation, drugs, autoimmune diseases, and other primary glomerulopathies. The pathophysiologic factor linking these secondary causes is endothelial cell injury. Frequently, more than one secondary cause may coexist, and it may be difficult to be certain of the main cause. Furthermore, as noted above, secondary causes may merely be triggers in those with underlying genetic susceptibility, i.e., having the same mutations associated with primary complement-mediated aHUS. Adjudication of such cases may be difficult, i.e., primary complement-mediated TMA with an associated trigger versus secondary TMA with underlying genetic predisposition (10). The chance for relapse or recurrence after transplantation would seem to be higher in the former, as long as the trigger could be removed in the latter.

Listed among rare secondary causes of TMA is the presence of a paraprotein, associated either with a definitive malignancy, such as myeloma or lymphoplasmacytic lymphoma, or with a lesser burden of plasma or B-cells falling below criteria for frank malignancy. Treatment of the underlying clone (B-cell or plasma cell) would be warranted, if the paraprotein is causing the TMA. Although not directly deposited in the kidney, the paraprotein may cause endothelial injury by disordering complement regulation at the cell surface. Similar such interference with complement regulation may also occur in the fluid phase and result in glomerulonephritis, referred to as C3G, including dense deposit disease (DDD) and C3 glomerulonephritis (C3GN).

Whereas C3G results from disordered regulation of the CAP in the fluid phase or at tissue surfaces lacking anchored regulators (29), primary complement-mediated aHUS results from abnormal endothelial cell-surface CAP regulation (30). Both C3G and aHUS are caused by mutations in the same CAP regulatory proteins. CFH has been the most studied regulator. Mutations leading to complete absence or those resulting in a lack of complement regulatory activity are generally situated at the N-terminus, result in dysregulation of the fluid phase, and present phenotypically as a C3G (31). In contrast, mutations clustering in the C-terminal region do not impair fluid-phase regulation, exhibit defective cell-surface regulation, and present as complement-mediated aHUS (28, 31). However, it is not always so clear cut, as the same mutation in family members may produce alternate phenotypes (C3G or aHUS) (32), and the phenotype may change following transplantation (33, 34) or even during the course of disease in a given patient (35, 36). Furthermore, both aHUS and C3G may coexist simultaneously on a given biopsy (37). Hence, insight into the pathophysiology and potential treatment of aHUS caused by disordered CAP regulation and the linkage to paraproteins can be gleaned from study of C3G patients.

The C3Gs, both DDD and C3GN, have been associated with paraproteins for several decades. In 1992, Meri et al. described a case of hypocomplementemic (low C3) membranoproliferative GN associated with a monoclonal paraprotein (λ-dimer) demonstrating isolated complement deposition in the absence of immunoglobulins on IF that today would be clearly called C3GN (38). This dimer was shown to interact directly with CFH resulting in activation of the CAP in a dose dependent manner. It did not bind to C3bBb and would not be considered a C3 nephritic factor. Further characterization of the dimer revealed binding to the short consensus repeat domain 3 of CFH, thereby inhibiting binding to C3b (39).

Several case series subsequently highlighted the association of paraproteins with C3G. Bridoux et al. studied 6 French adults with C3G and associated paraproteins, 5 being MGUS. No mutations in complement regulatory proteins were found. Five of 6 progressed to ESKD (40). Zand et al. from the Mayo Clinic studied 32 cases with C3GN and found 10 (31%) had paraproteins, 5 of which were MGUS, and 3 had no detectable clones (41). Ravindran et al. expanded on this work from the Mayo Clinic to total 36 patients with C3G, (including 4 with DDD) of which 26 had an MGUS, 1 cryoglobulinemia, and the rest malignancy (myeloma or CLL) (42). Of 21 patients with genetic testing, only 2 had any mutations (one heterozygous C3 and the other heterozygous CFHR5). Additionally, 34% had low C3 levels and 46% had C3 nephritic factors. Of the 16 patients receiving clone-directed therapy, 10 achieved a hamatologic response, and 7 of those 10 had a renal response.

Chauvet et al. retrospectively analyzed 50 patients with C3G and a paraprotein from the French registry of C3G, including 30 with MGUS, 17 with myeloma, and 3 with CLL (37). C3 was low in 43%, 6% had C3 nephritic factors, 19 had anti-CFH antibodies, and soluble C5b-9 was elevated in 79% of tested patients. For treatment, 29 received clone directed chemotherapy (bortezomib in 22), 8 received immunosuppression, and 13 received symptomatic therapy alone. Of the 29 patients given clone-directed chemotherapy, 17 (59%) achieved hematologic response, and 15 of those 17 (83%) had a renal response. Only 1 of 21 (5%) given general immunosuppression or conservative therapy had a renal response. However, 5 patients given chemotherapy had serious adverse events that were fatal in 3 cases. Notably, 8 of the 50 (16%) had concurrent TMA and C3G on their biopsies.

TMA has also been associated with monoclonal gammopathy, although less so compared to C3G. In patients with frank malignancy-associated paraproteinemia (e.g., myeloma), TMA has been reported (43–46) and may result from therapy as well (47). Proteosome inhibitors have been shown to cause TMA (48–50), and hematopoietic stem-cell transplantation (HSCT) is a well-known secondary cause of TMA, even if autologous (51, 52).

In addition to our 3 patients, others have reported TMA with underlying MGUS, including several case reports (53–57). In the largest case series, Ravindran et al. retrospectively analyzed 146 patients from the Mayo Clinic with MAHA or biopsy proven TMA that had been screened for paraproteins and found that 20 patients (13.7%) were positive, including 21% of those over 50 years old (58). The incidence of paraproteinemia with TMA was thus 4 times the incidence of paraproteinemia in the general population. Eleven of the 20 were tested for ADAMTS13 levels, and 2 were diagnosed with TTP (ADAMTS13 activity ≤ 10%). The remaining 9 were considered aHUS, and the 9 patients untested for ADAMTS13 activity were considered unclassifiable, since TTP was not ruled out. Of the 20 patients, 15 had MGUS, 2 myeloma (1 smoldering), 2 POEMS, and 1 T-cell leukemia. Infection was the precipitating cause in 3, cyclosporine in 1, and the others were considered idiopathic. Genetic studies were not performed. Treatment was variable, including plasma exchange (11 patients), steroids, immunosuppression, and/or clone directed therapy. Ten progressed to ESKD.

Yui et al. described 9 cases collected from around the United States with paraprotein-associated TMA, including 5 with myeloma, 1 with lymphoplasmacytic lymphoma, and 3 with MGUS (hence representing MGRS) (59). Only 4 exhibited MAHA. Only 1 was tested for ADAMTS13 activity (normal), and only 2 had genetic analysis.

Our 3 cases add to this small list of reported cases of MGRS presenting as aHUS and they have been analyzed for secondary causes and underlying genetic mutations. Similar to the relationship between infection and TMA, paraproteins may be either a trigger in those with underlying susceptibility or the sole cause of the process. Additionally, paraproteins could serve as underlying susceptibility factors that require a “second hit”, similar to genetic mutations. Given the relatively transient nature of the clinical kidney manifestations of our first 2 patients (normalization of proteinuria), an infection that resolved seems most likely as a secondary cause. It seems improbable that the kidney TMA would remain active with complete normalization of proteinuria that was previously significantly elevated and was the main reason for biopsy in both cases, and hence kidney biopsies were not repeated. The slight creatinine increases are explainable as functional (hemodynamic) effects of intensified BP control and maximal RAS inhibition.

Therapeutic options for paraprotein-associated TMA include clone directed therapy to eliminate the paraprotein and/or C5 inhibition to inhibit complement activation. Whereas clone directed treatment of MGRS has not been shown to prolong life by preventing progression to overt malignancy, hematologic remission may induce a kidney remission that delays or prevents progression to ESKD. Persistence of a pathogenic paraprotein following kidney transplantation can result in recurrent allograft disease. Hematologic remission prior to transplantation would likely prevent recurrence, and this represents a strong indication for clone directed therapy in any transplant candidate. If a patient is not a transplant candidate and the kidney disease is far advanced, clone directed therapy is probably not indicated from the kidney perspective. We initiated clone directed therapy for 2 of our patients due to the obvious plasma cell clone in each case and the chronicity of the kidney disease. The third patient had no detectable clone, no chronic changes on biopsy, and a resolving clinical picture. The decision was made to not immediately treat this patient, but to follow closely for any clinical recurrence.

The role of C5 inhibition in paraprotein-associated TMA remains uncertain. Eculizumab and ravulizumab now approved for therapy of aHUS with or without detectable mutations. Whereas the presence of an underlying genetic mutation did not significantly affect the chance of responding to eculizumab, the presence of a mutation may enhance the chance for relapse upon discontinuation (60). If complement system dysregulation is pathogenic in paraprotein-related TMA, then C5 inhibition is likely beneficial. If such dysregulation is in fact mediated by the paraprotein, then hematologic remission induced by clone specific therapy may be sufficient. If hematologic remission is unobtainable, the TMA progresses despite hematologic remission, or the disease is life-threatening at presentation, C5 inhibition should be considered. The presence of underling genetic mutations or at-risk polymorphisms would bolster support for using C5 inhibition, but they are not required. Complement involvement, as evidenced by low serum C3 and/or C4, elevated soluble C5b-9, or positive biopsy staining for C5b-9 lends further support.

Complement system activation has been studied in the major categories of TMA, including both primary and secondary, in addition to complement-mediated aHUS. Both TTP and STEC-HUS (both considered primary TMAs) show evidence of complement activation (61), and anecdotal reports indicate a benefit to eculizumab in refractory cases of either STEC-HUS (62) and TTP (63). In a retrospective, multi-center analysis of 75 patients with STEC-HUS, pathogenic variants with minor allele frequency (MAF) < 0.1% were more common versus either 80 French controls or 503 individuals from the 1000 Genomes Project; however, pathogenic variants with a higher MAF (< 1%) were not significantly more common (64).

Mutations in genes controlling CAP activation have also been found to a variable degree underlying secondary causes of HUS, depending to a large extent on the specific cause. For example, malignant hypertension-associated HUS (65–67), pregnancy associated aHUS (68), and *de novo* TMA following transplantation have been shown to have a high frequency of underlying mutations, whereas drug induced-TMA (11, 22) and autoimmune disease-associated HUS (11, 22) do so much less frequently. The use of C5 inhibition in secondary TMA is of uncertain benefit with conflicting data published to date. Again, this may be determined by the specific underlying cause.

Cavero et al. evaluated 29 patients drawn from 11 Spanish hospitals diagnosed with secondary HUS (15 drug-induced, 8 with autoimmune systemic illness, 2 post-partum, 2 cancer-related, and 2 other) that were given eculizumab (69). All 29 had severe MAHA and renal impairment, with 14 needing dialysis. Only 2 of 22 tested patients harbored underlying pathogenic variants of complement regulatory proteins. Overall, 20 of 29 responded to eculizumab with complete resolution of MAHA and ≥ 25% reduction of serum creatinine.

In contrast, Le Clech et al. studied 110 patients with secondary HUS referred for complement analysis to the French national registry of patients with HUS (22). The major secondary causes included drugs (29%), autoimmune diseases (24%), infections (17%), malignancy (10%), glomerulonephritis (9%), and extra-renal solid-organ transplantation (8%). The frequency of pathogenic, rare (MAF < 0.1%) complement gene variants was not significantly different compared to 80 French or 503 European controls. There was no difference between patients with secondary HUS and healthy donors in the frequency of the at-risk CFH haplotype tgtgt or in the prevalence of the CFHR1-3 deletion, but the at risk MCP haplotype ggaac was significantly more prevalent in those with disease (17% versus 6%, p = 0.04). Overall, 38 patients were given eculizumab, with no difference in hematologic remission, development of chronic kidney disease stages 3/4 or 5, or death as compared to untreated patients. Although the 38 given eculizumab were sicker, when matched to 38 patients not given eculizumab based on age, creatinine, platelet count and hemoglobin, renal outcome at 3 months was not different.

Based on these conflicting data, the appropriate use of C5 inhibition in secondary HUS remains to be determined. No data exist regarding its use in paraprotein-associated HUS other than isolated case reports (53, 56). Schurder et al. reported a case of paraprotein-related TMA that failed to have a kidney response to plasmapheresis, despite hematologic improvement that subsequently had near normalization of kidney function with eculizumab (56). In contrast, Cheungpasitporn et al. reported a patient with an MGUS and biopsy-proven TMA accompanied by MAHA that was refractory to eculizumab, but responsive to clone-directed therapy (bortezomib, lenalidomide, dexamethasone) (53). Similarly, Mahmood et al. described a patient with MAHA, progressive renal dysfunction, kidney biopsy showing a MPGN pattern with microthrombi in glomerular capillaries, and an associated IgAλ paraprotein (57). Clone directed therapy markedly ameliorated the MAHA and improved kidney function. In our opinion, clone-directed therapy is the preferred treatment in such cases. We would consider C5 inhibition if hematologic remission did not occur with clone directed therapy, the TMA remained active after obtaining hematologic remission, and/or severe, life-threatening disease was present.

## Conclusion

Three cases with benign paraprotein-associated, biopsy-proven, kidney limited TMA are presented. In our opinion, this is more than a chance association of an MGUS and TMA, and the paraproteins are most likely causative, thereby fulfilling criteria for MGRS. The approach to such case should entail evaluation to rule-out underlying B-cell lineage malignancy that would require clone-directed therapy. TTP, STEC-HUS, and all secondary causes of TMA should also be ruled out with careful assessment for associated MAHA. Genetic analysis for underlying CAP mutations and at-risk haplotypes should be obtained along with other evidence of complement involvement, including serum levels of C3, C4, C5b-9, and C3 nephritic factors. The biopsy should be evaluated for C5b-9. Even in the absence of malignancy, the underlying clone should be identified, if possible, with the intent of clone-directed therapy, especially if the kidney function is preserved and/or the patient is a transplant candidate. C5 inhibition should be strongly considered if severe, life-threatening disease involving other organs is present, bolstered by genetic mutations and other evidence of CAP involvement. We would also consider C5 inhibition if hematologic remission cannot be obtained or the TMA remains active after such remission.

## Author Contributions

EF reviewed the literature and wrote the first and final drafts of the paper. EN, LL, RG, and JF provided critical review and contributed to the writing of the final draft. All authors contributed to the article and approved the submitted version.

## Conflict of Interest

The authors declare that the research was conducted in the absence of any commercial or financial relationships that could be construed as a potential conflict of interest.

## Publisher’s Note

All claims expressed in this article are solely those of the authors and do not necessarily represent those of their affiliated organizations, or those of the publisher, the editors and the reviewers. Any product that may be evaluated in this article, or claim that may be made by its manufacturer, is not guaranteed or endorsed by the publisher.
